# Clinical Sphingolipids Pathway in Parkinson’s Disease: From GCase to Integrated-Biomarker Discovery

**DOI:** 10.3390/cells11081353

**Published:** 2022-04-15

**Authors:** Ali Esfandiary, David Isaac Finkelstein, Nicolas Hans Voelcker, David Rudd

**Affiliations:** 1 Drug Delivery, Disposition and Dynamics, Monash University, Parkville, VIC 3052, Australia; ali.esfandiary@monash.edu (A.E.); nicolas.voelcker@monash.edu (N.H.V.); 2 Melbourne Centre for Nanofabrication, Victorian Node of the Australian National Fabrication Facility, Clayton, VIC 3168, Australia; 3 Howard Florey Institute of Neuroscience and Mental Health, Parkville, VIC 3052, Australia; david.finkelstein@florey.edu.au; 4 Commonwealth Scientific and Industrial Research Organization (CSIRO), Clayton, VIC 3168, Australia; 5 Materials Science and Engineering, Monash University, Clayton, VIC 3168, Australia

**Keywords:** Parkinson’s disease, sphingolipid pathway, glucocerebrosidase1, diagnosis

## Abstract

Alterations in the sphingolipid metabolism of Parkinson’s Disease (PD) could be a potential diagnostic feature. Only around 10–15% of PD cases can be diagnosed through genetic alterations, while the remaining population, idiopathic PD (iPD), manifest without validated and specific biomarkers either before or after motor symptoms appear. Therefore, clinical diagnosis is reliant on the skills of the clinician, which can lead to misdiagnosis. IPD cases present with a spectrum of non-specific symptoms (e.g., constipation and loss of the sense of smell) that can occur up to 20 years before motor function loss (prodromal stage) and formal clinical diagnosis. Prodromal alterations in metabolites and proteins from the pathways underlying these symptoms could act as biomarkers if they could be differentiated from the broad values seen in a healthy age-matched control population. Additionally, these shifts in metabolites could be integrated with other emerging biomarkers/diagnostic tests to give a PD-specific signature. Here we provide an up-to-date review of the diagnostic value of the alterations in sphingolipids pathway in PD by focusing on the changes in definitive PD (postmortem confirmed brain data) and their representation in “probable PD” cerebrospinal fluid (CSF) and blood. We conclude that the trend of holistic changes in the sphingolipid pathway in the PD brain seems partly consistent in CSF and blood, and could be one of the most promising pathways in differentiating PD cases from healthy controls, with the potential to improve early-stage iPD diagnosis and distinguish iPD from other Parkinsonism when combined with other pathological markers.

## 1. Introduction

Parkinson’s disease (PD) is the second most prevalent progressive neurodegenerative disorder with no definitive diagnosis available for idiopathic cases (iPD), which accounts for the majority of cases (85–90%) [1]. Currently, iPD diagnosis relies on changes in motor symptoms: bradykinesia (slowness of movement) along with rigidity (increase in muscle tone) or resting tremor [2]. Unfortunately, diagnosis is only ~80% accurate even with a lifetime of living with the condition. This proportion is even lower in people in the first years after diagnosis [3,4]. Clinical symptoms are now being thought of as a sign of advanced disease and as a consequence of long-lasting disruptions in underlying cellular mechanisms. Clinical diagnoses using motor symptoms are observable only after 60–80% of dopaminergic neurons are lost in the substantia nigra (SN) [5]. These limitations are problematic for clinical trials and the implementation of new therapies with the aim of neuroprotection. Research is starting to now focus on the underlying alterations in cellular mechanisms preceding neuronal loss with the hope that these could be detected in the prodromal (before the onset of motor symptoms) or premotor stages of PD.

Antemortem iPD diagnosis is urgently required as, currently, the definitive diagnosis of iPD is available only postmortem by confirmation of neuronal loss in the SN together with the presence of Lewy bodies [6]. The prodromal stage has been receiving much attention as it is being recognized that a very high proportion of people with a cluster of prodromal symptoms—rapid eye movement sleep behavior disorder (RBD), gastrointestinal problems, loss of the sense of smell, changes in color vision, erectile dysfunction [7]—go on to develop PD and parkinsonism. This correlation has fostered excitement as metabolic changes occurring during the prodromal phase could be potential biomarkers for differentiating iPD from other similar disorders and confounding symptoms from PD-specific symptoms. Attempts at non-invasive and antemortem diagnosis of iPD have introduced imaging techniques as a useful tool in iPD diagnosis. Though imaging techniques are not definitive for PD diagnosis, they still could enhance the diagnostic accuracy by excluding non-degenerative conditions (dopamine transporter or vesicular monoamine transporter 2 and optical coherence tomography of the retina [8,9]), distinguishing PD from secondary parkinsonism or atypical parkinsonism (structural MRI with 50% sensitivity), or contributing in PD differentiation from multiple system atrophy or progressive supranuclear palsy (diffusion-weighted-MRI, fluorodeoxyglucose PET, transcranial ultrasound) [1].

Other novel imaging techniques are emerging as potential tools for the prodromal diagnosis of iPD. For instance, neuromelanin imaging MRI and quantitative susceptibility mapping [10,11,12] differentiate PD from healthy controls with around 80% sensitivity and specificity. The accuracy is more promising in dorsal nigral hyperintensity techniques that offer a pooled sensitivity of 98% and specificity of 95% [13]. Functional examination of striatal dopaminergic neurons using radionuclide tracers in the dopamine transporter—SPECT is another imaging technique used for differentiation of atypical parkinsonism from PD; nevertheless, it is not suggested for differential diagnosis of neurodegenerative parkinsonism [14]. Altogether, these novel techniques are based on nigral pathology and they could be specific to neurodegenerative parkinsonism and not merely PD. Imaging techniques are not an ideal approach for prodromal iPD diagnosis as they are mostly based on irreversible neurodegeneration in the iPD brain, though identifying the loss of striatal vesicular monoamine transporter 2 with imaging may signal the transition from prodromal to the symptomatic phase of iPD [15].

Neuronal loss is preceded by long-lasting alterations in cellular mechanisms, the metabolites of which could be identified for prodromal diagnosis of iPD. Since the initial discovery that familial parkinsonism can be caused by multiplications and mutations in the SNCA gene, there has been a worldwide focus on the role of α-synuclein (αSyn) in the progression of PD [16]. Indeed, PD and αSyn are inextricably linked in the pathogenesis of PD. The “gold standard” pathological diagnosis of PD is postmortem confirmation of αSyn aggregation in the form of Lewy bodies with the nigra–pinpointing inadequate protein clearance system functioning. 

Among pathways implicated in PD, the dysfunction of the protein clearance system is the closest cellular mechanism that could explain why misfolded protein accumulation in the brain appears as a core pathology in PD. The accumulation of intra-cellular αSyn implicates protein clearance systems; the ubiquitin-proteasome system [17] and the autophagy-lysosome pathway [18,19,20] in the pathology of the disease. αSyn is an abundant presynaptic protein that normally associates the lipid membranes of the presynaptic vesicles [21] and modulates neurotransmitter release [22]. An intact membrane is essential for small extracellular vesicle-induced modulation of αSyn fibrillization [21,23]. The chemical properties of lipids strongly affect the kinetics of the vesicle-membrane-induced aggregation of αSyn [23]. Impaired sphingolipid metabolism in PD is linked to the protein clearance system dysfunction in a bidirectional way [24,25]. This is further supported by the inhibitory impact of mutant GBA on the chaperone-mediated autophagy-lysosomal pathway (CMA) and, thereafter, αSyn accumulation [26].

The autophagy-lysosomal pathway could be one of the promising pathways in differentiating PD from controls. This pathway orchestrates lipid metabolism, given the fact that lysosomes host multiple lipid hydrolysis enzymes and act as the hub for the degradation of various types of macromolecules [27]. Lipids have heterogeneous classes including glycosphingolipid glucosylceramides, are involved in membrane organization and lipid rafts formation, adhesion, growth, differentiation development and inflammation [28], and are key players in PD pathogenesis [29].

One of the most common risk factors for iPD is mutations in the glucosylceramidase beta (GBA) gene, which is associated with early disease onset, severe cognitive dysfunction and a higher mortality rate from diagnosis compared to noncarriers [30,31,32]. The GBA gene encodes the glucosylceramidase (GCase) enzyme and its homozygote mutations are the cause of Gaucher disease [33]. The GCase breaks glucosylceramide down to glucose and ceramide in the late endosomes and lysosome. This is referred as the salvage pathway of ceramide synthesize, which interchanges with other ceramide synthesis pathways: the de novo ceramide synthesis and sphingomyelinase pathway (as reviewed in [34]).

Since PD and the GBA gene association were reported in 1996, many studies have explored the phenotypic impact of GCase activity in PD, yet data remain inconclusive [35,36,37]. The availability of comprehensive and precise analytical methods (lipidomics and proteomics) for measuring multiple factors at once initiated a growing interest in holistic monitoring of alterations in sphingolipid metabolism for differentiating iPD [37,38,39,40,41]. The development of data integration methods enabled combining metabolomics data with other diagnostic data (e.g., genomics data) that is equipped for systems biology approaches, where the impact of other pathways on iPD pathogenesis could be modelled for biomarker purposes [42,43]. Here, by focusing on the GBA, we will review the current literature supporting the diagnostic potential of sphingolipid pathway dysregulation, in the human brain and biofluids, and their integration with semi-sensitive biomarkers and putative pathological pathways for the development of an iPD specific signature.

## 2. Protein Clearance System Impairment in PD-Ubiquitin-Proteasome System (UPS)

For misfolded proteins to be cleared, ubiquitin ligases are required to tag them with ubiquitin molecules to signal proteolysis. These ubiquitylated proteins are then handed over to the 26S proteasome—the most used proteasome in mammals with a 20S catalytic core and 19S regulatory particles at both ends that break misfolded proteins into small peptides. Non-proteasomal endopeptidases and aminopeptidases then digest these small peptides into monomeric amino acids for reused in new protein synthesis [44].

**Figure 1:**
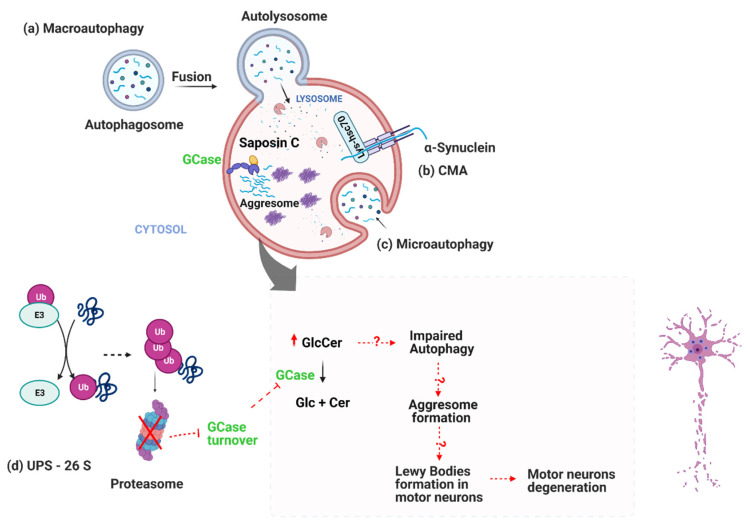
Disrupted protein clearance systems (autophagy-lysosome pathway and ubiquitin-proteasome pathway) in PD motor neurons and GCase dysfunction. (**a**) Macroautophagy. (**b**) Chaperone mediated autophagy (CMA) is a more selective process and targets KFERQ amino-acid-motif containing proteins such as αSyn. (**c**) Microautophagy or pinocytosis of intracellular contents. (**d**) Defective ubiquitin-proteasome system (UPS) failure in eliminating misfolded proteins tagged with ubiquitin such as GCase precede aggresome formation and neurodegeneration. Defects in GCase turnover [46] lead to glucosylceramide (GlcCer) accumulation, which later contributes to αSyn aggregation and impaired autophagy [25]. Altogether, impaired protein-clearance systems cause neurodegeneration in PD (created with BioRender.com. accessed on 1 January 2022). Events leading to GCase dysfunction and its consequences are indicated by red-color arrows. Dashed arrows represent a series of reactions, and dashed arrows with the question mark in the middle indicate unknown mechanisms.

Failures in the ubiquitin-proteasome system (UPS) in PD are reported in both familial and iPD cases. In familial cases, an E3 ubiquitin ligase, parkin, is associated with early-stage PD (<30 years) [45]. Interestingly, another member of E3 ubiquitin ligase, thyroid hormone receptor-interacting protein 12 (TRIP12), which accumulates in sporadic PD brains, degrades GCase enzymes and controls their turnover (Figure 1) [46]. Postmortem analysis of nigral tissues has shown a 30% deficiency in proteasome enzymatic function in iPD cases compared to healthy controls [47]. Though the mechanism of this proteasome enzymatic dysfunction is unknown, the selective loss of 20S core α-subunits restricted to dopaminergic neurons of the SN in sporadic PD could be a possible explanation [17]. Taken together, UPS dysfunction in iPD has the potential to explain the pathologic mechanism underlying protein accumulation and Lewy bodies formation in the PD brain, however, more questions are yet to be addressed.

## 3. Protein Clearance System Impairment in PD-Autophagy-Lysosome Pathway Fails in Clearing Sphingolipids

Autophagy is a conserved lysosomal degradation process for selective removal of damaged subcellular organelles (mitochondria, peroxisomes, endoplasmic reticulum), midbody ring structures, ribosomes and aggresomes [48]. This process requires lysosomes to remove unwanted material in a so-called autophagy-lysosome pathway (Figure 1) and has a critical role in PD pathology (as reviewed by [49]). Indeed, some PD-related genes (SNCA, VPS35, Parkin, PINK1, FBXO7, ATP13A2 and GBA) are involved in autophagy processes and their abnormal levels are implicated in the PD brain (as reviewed in [50]). Lysosomes are hosting multiple hydrolyses as the ultimate hub for the degradation of various types of macromolecules including lipids and, as such, the autophagy-lysosome pathway is involved in orchestrating lipid metabolism [27]. Groups of lipids such as eicosanoids, endocannabinoids, oxysterols, fatty acids and sphingolipids with cell-regulatory characteristics are classified as bioactive lipids and deemed as key players in PD pathophysiology (as reviewed in [51]). Sphingolipids are a member of bioactive lipids with a reciprocal effect on the autophagy process in PD. Accordingly, defective sphingolipids pathways are reported through a few clinical studies on PD subclasses (Table 1).

Sphingolipid pathway impairment is not restricted to only PD neurons or PD CSF but seems to be a systemic feature of PD evidenced by reduced GCase activity in blood and fibroblasts [63], which are invaluable for non-invasive biomarker purposes. The GCase activity is pathologically informative in familial PD (PD-GBA) and research is underway to elucidate its diagnostic potential in iPD.

### 3.1. Is GCase Activity a Potential Biomarker for iPD?

The GCase enzyme is a 536 amino acid length lysosomal-membrane-anchored protein that catalyzes glucosylceramide into glucose and ceramide [64], and glucosylsphingosine into glucose and sphingosine [65]. Mutations in the GBA gene are implicated in different types of Gaucher disease (GD) (type 1, 2 and 3) and PD. In GD, homozygous mutations in the GBA gene cause insufficient activity of GCase, which is the gold standard marker for GD diagnosis [33,66]. Heterozygous mutations in GBA that cause haploinsufficiency in GCase activity are the greatest risk factor for PD [31,67]. Across different studies, 5–25% of iPD are carriers of these mutations, which are associated with a 5–20-fold increase in developing PD compared to non-carriers [31,68]. There are some differences in GBA mutations for GD and PD. For instance, some GBA alterations that are considered “mild mutations” and do not cause GD (E326K (p.E365K) and T369 M (p.T408M), still predispose carriers to parkinsonism [69,70]. GBA heterozygous mutations are clinically associated with early-onset PD with severe cognitive symptoms [31,67]. PD age of onset correlates with GBA mutation status and PD cases with homozygous mutations manifest the disease approximately 6–11 years earlier than in heterozygotes; however, the overall PD incidence is comparable [71].

**Figure 2:**
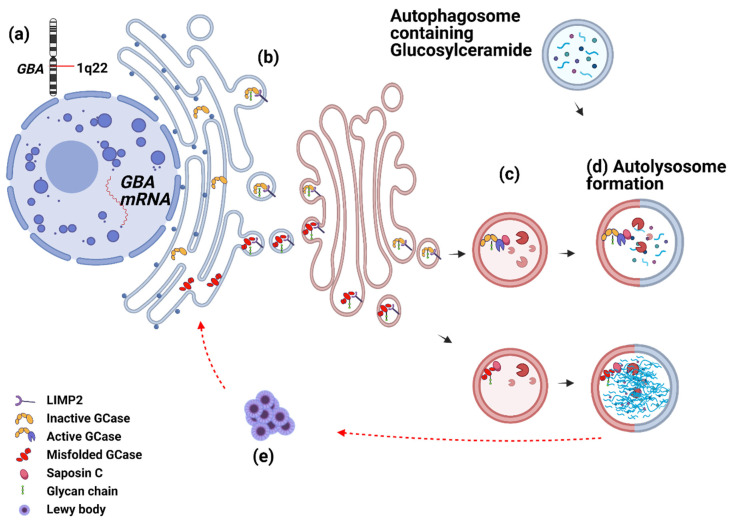
GCase from birth to death. (**a**) Chromosome locus of GBA gene with 14 exons. More than 300 GBA variants are associated with GD and around 130 mutations are associated with PD [76]. (**b**) After GBA is transcribed into mRNA, it becomes synthesized in the ER and undergoes post-translational modifications, such as glycosylation [65]. The GCase enzyme attaches to LIMP2 and is guided to the Golgi and lysosomes. LIPM2 levels in SN of PD-GBA and iPD do not change [36], but levels are reduced in iPD fibroblasts and correlate with reduced GCase activity [63]. (**c**) After glycosylated-GCase reaches the lysosome it becomes activated by saposin C. (**d**) Activated GCase clears glucosylceramides engulfed inside the autophagosome; however, the incorrect conformational state of GCase and its deficient activity cause glucosylceramide accumulation in the lysosomes. (**e**) There seems to be a bidirectional effect between glucosylceramide accumulation and Lewy body formation. The presence of Lewy bodies is suggested to disrupt correct conformational states and trafficking of GCase proteins towards lysosomes [25] and vice versa, implicating the mutant GCase in αSyn aggregation [26,77]. (Created with BioRender.com. accessed on 22 December 2021).

In definitive PD cases, which are diagnosed by postmortem confirmation of αSyn accumulation into Lewy bodies, reduced GCase activity is reported in both GBA mutation carriers and iPD [35,36]. In these independently conducted studies on the SN tissues of PD-GBA wild type cases, the GCase enzyme catalytic activity is reduced as well as its mRNA and protein levels [35,36,72]. There is strong and emerging evidence that mutations in the GBA gene can cause PD and, additionally, that GCase activity is altered in idiopathic disease without mutation. The mutation frequency in healthy cases is still lower than in PD. In a study with 79 PD and 61 healthy controls, 4.9% of controls and 12.7% of PD are GBA mutation carriers [73]. In another study conducted in Australia, 18.75% of 48 PD and 6.8% of 44 healthy controls are GBA mutation carriers [62]. These data suggest that GCase reduced activity is not merely due to mutations and other mechanisms regulating GCase trafficking, and activation should be explored (Figure 2). Due to the prognostic value of reduced GCase activity and its implication in definitive iPD, several studies asked the question of whether GCase could be a potential biomarker for iPD diagnosis (Table 1) by looking at different biospecimens; however, the answer remains inconclusive. Analysis of CSF specimens, that interchange with interstitial fluid in the brain, has indicated GCase activity as non-informative [37,60,61], while others associate reduced GCase activity with PD irrespective of GBA mutation status [61]. Blood interchanges with CSF through the blood–CSF barrier and is expected to be a partial proxy for detecting changes in the brain and CSF. Data coming from blood samples (serum, plasma and dried blood spots) are also inconsistent. Though GCase activity is reduced in the serum of PD-GBA and PD-GBA wild-type [37], in dried blood samples, for example, reduced GCase activity is not necessarily associated with PD risk [74]. This discrepancy could be due to the subjective nature of the clinical diagnosis of selected PD cases for analysis, biospecimen sourced (monocytes versus dried blood samples) and different strategies in evaluating the GCase activity (as reviewed by [75]). Altogether, though PD diagnosis by GCase activity in isolation remains inconclusive, its prognostic value holds the hope for increasing diagnosis accuracy by incorporating GCase with upstream or downstream regulators such as saposin C (SapC) and post-translational modifications. This would provide stronger evidence for an altered pathway, from GCase transcription to activity, and be less reliant on a single marker.

#### 3.1.1. GCase from Birth to Death—What Can Go Wrong?

GCase enzyme activity depends on several regulatory mechanisms including structure-functional changes, pH and the availability of its activator SapC; disruptions in any of these steps could potentially affect downstream GCase activity. The GBA gene located at 1q21 encodes the lysosome-membrane-anchored GCase enzyme and its mutations are implicated in iPD [66]. After the GCase protein is synthesized in the endoplasmic reticulum (ER) it becomes glycosylated (particularly at Asn19), which is deemed crucial for its catalytic activity [78]. One possible mechanism for the reduced downstream activity is the disruptions in the structure-functional changes of GCase impacting intercellular trafficking, interaction with SapC and substrate stability, all of which could underlie GCase loss-of-function in wild type carriers (Figure 2, [24]). Currently, 20–30% of carrier diagnoses in GD by GCase activity are false positives and false negatives and introducing an activity-dictating structural pattern for GCase could also be useful for GD carrier diagnosis [33]. To the best of our knowledge, there is no currently published evidence exploring the impacts of GCase structural changes on catalytic activity in iPD and further studies are warranted.

#### 3.1.2. Saposin C and GCase

The prosaposin gene (PSAP) encodes a precursor protein that forms into sphingolipid activator proteins (SAPs)—saposin A-D—that facilitate lysosomal hydrolysis of sphingolipids [79]. In lysosomes, SapC interaction with GCase alters GCase catalytic activity by inducing conformational changes [80]. Furthermore, this interaction stabilizes the GCase enzyme against protein clearance systems [81]. Accordingly, among many mutations reported in GBA, two common and clinically relevant mutations in GD, N270S and L444P, are suggested to destabilize the SapC and GCase complex [82]. Full sequence analysis of PSAP in patients suffering RBD—a sign of the prodromal stage of synucleinopathies such as PD [83]—report an association of three different and rare mutations with idiopathic rapid eye movement sleep behavior disorder [83]; however, full sequencing of PSAP was not found to fully support a role for PSAP or SapC in PD patients [84]. Regardless of the current argument on the contribution of PSAP mutations to PD, its impaired function could be indirectly inferred from GCase function, ideally from GCase substrates levels as its activity analysis in isolation could be impacted by external factors.

#### 3.1.3. Do GCase Substrates Differentiate iPD?

The inconsistency of data on GCase activity is also represented at the substrate level. Here, the conflict starts from brain data, with some reports of no accumulation of GCase substrates (glucosylceramide and glucosylsphingosine) in the PD-GBA brain [41], yet the accumulation of glucosylsphingosine has been seen in specific age groups of iPD [59]. However, in a recent study on CSF, a high level of glucosylceramides is reported [37]. Though these limited data on various brain and CSF specimens do not support a direct association between glucosylceramide level and PD [85], further studies on a broader scale are warranted.

The GCase enzyme interacts with sphingolipid pathways and changes in specific substrates could have variable indications in PD diagnosis; therefore, there is growing interest in a comprehensive analysis of sphingolipids pathway for biomarker discovery.

## 4. Sphingolipids in PD Diagnosis

Post-mitotic neuron survival is partly governed by sphingolipids through modulating the autophagy process (as reviewed by [86]). Autophagy eliminates aggregated proteins and its impairment is reported through a few clinical studies on PD subjects [18,19,20]. Sphingolipids are becoming linked to the core pathological feature of PD, αSyn aggregation. This is specifically supported by a recently proposed bidirectional link between GCase deficiency and Lewy body formation [25,26]. Though the exact molecular mechanism is still unidentified, the data is in line with the role sphingolipids play in neuronal survival, exemplified by the monosialoganglioside GM1. GM1 is a member of glycosphingolipids that binds and assists in αSyn structural stability and its deficiency has been associated with αSyn aggregation and neurodegeneration [87,88]. GM1 and αSyn interactions are supported through several clinical studies. GM1 level deficiency in the PD brain is not only restricted to SN, but it is also reduced in the less affected part of the PD brain (occipital cortex), and systemically in CSF and blood across different clinical studies (Table 1). Interestingly, the level of GM1′s metabolic precursor, GD1a [89], shows the same changing trend in PD tissues (Table 1). The lower GM1 and its precursor levels could predispose dopaminergic neurons to degeneration since it is essential for proper glial cell-derived neurotrophic factor (GDNF) signalling that protects dopaminergic neuron survival [57].

Ceramides are the precursor of complex sphingolipids, which are implicated in PD pathology (as reviewed in [34]) and cognitive decline [90], nevertheless, data on the diagnostic potential of ceramides is inconsistent across different studies on PD brain, CSF and blood (Table 1). This discrepancy could be explained by different GBA mutation status of patients and controls, sample processing (blood versus plasma), involvement of the tissue of study in PD pathogenesis (less involvement of occipital cortex compared to anterior cingulate) or the time of sampling (antemortem vs. postmortem CSF sampling). Therefore, further studies are required to standardize the diagnostic potential of ceramides.

**Figure 3:**
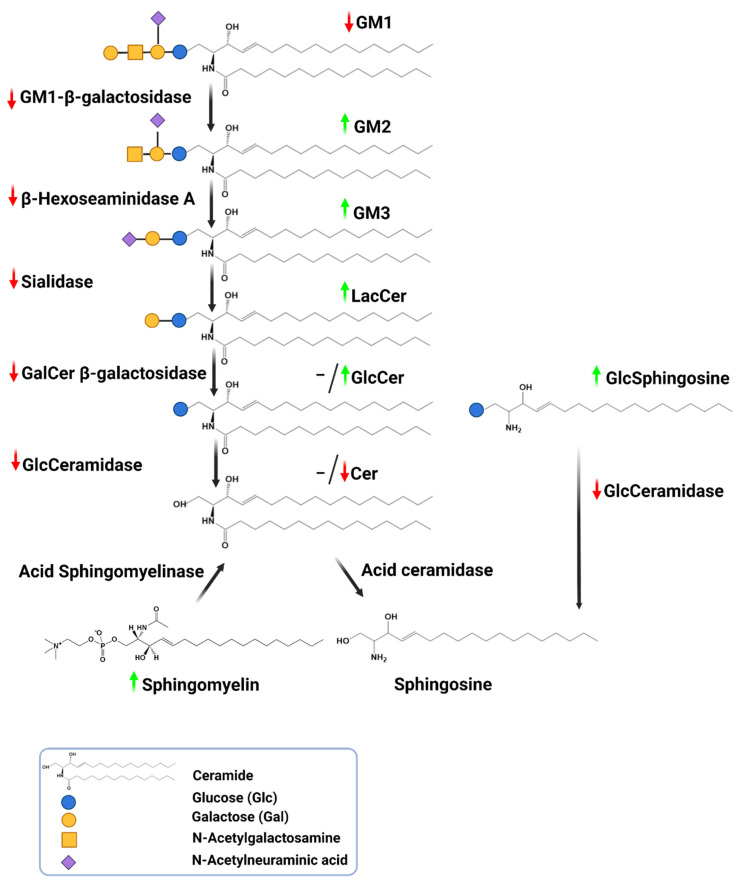
Evidence-based alterations in the sphingolipids’ catabolism pathway implicated in PD tissues. The overall changes show a decrease in the activity of the enzyme which is accompanied by increased levels of substrates [36,37,38,39,40,91]. Corresponding references for data, their percentage of changes and tissues of origin are contained in the Table 1. PD brain data are generated from definitive PD cases and, as such, they are considered more valuable than CSF or serum data in describing metabolic shifts. Green-colored arrow upward: increase; red-colored arrow downward: decrease; hyphen: no change. (Created with BioRender.com. accessed on 15 November 2021).

The data on other components of the sphingolipid pathway is also limited; however, reduced activity of the enzymes involved in this pathway, and in some cases, the accumulation of corresponding substrates, introduce a systemic disruption in sphingolipid metabolism in PD subjects (Figure 3, [36,37,38,39,40,91]). This pathway dysregulation in definitive PD is partially represented in peripheral biospecimens such as CSF and blood (Table 1). Here, GM1, GD1a levels, galactosidase activity, and to some extent, GCase activity and its substrate glucosylceramide, could successfully differentiate PD cases from healthy controls even by monitoring CSF and blood (Table 1). These data suggest that monitoring sphingolipids in CSF and blood, which are both available antemortem and have lower degrees of invasiveness, have the potential to increase iPD diagnosis.

Sphingolipids’ pathway dysregulation is becoming indicated in prodromal PD. The GBA gene mutations are listed as an intermediate-strength genetic factor for prodromal PD, where the total PD risk in mutation carriers at age 65 is 9 times higher (18% in PD versus 2% in age-matched controls) [92]. Interestingly, serum levels of the sphingolipids are also indicated in RBD cases, which are considered a sign of prodromal PD. A meta-analysis of 1280 RBD patients with an average follow-up of 4.6 years reports that 14.56% of RBD patients develop PD [93]. Though the GlcCer level is not changed, a significant reduction in the level of lactosylceramide, Gb3, Gb4, GM2, GM3, GM1a and GD1a is reported compared to healthy and PD controls [40].

Altogether, data on using sphingolipids pathway as a diagnostic tool are promising but limited, and future studies with large patient populations and stratified controls could help confirm the specificity of different parts of the sphingolipid catabolism pathway and their respective diagnostic value for iPD.

## 5. Would Integrating Biomarkers Make iPD Diagnosis Possible?

Integrating metabolites representing iPD pathophysiology with lower specificity and sensitivity could enhance the accuracy of iPD differentiation from other parkinsonism. To the best of our knowledge, there is no published evidence using this approach, yet preliminary data on biomarker integration support enhanced diagnostic accuracy. For instance, a combination of GCase, cathepsin D and β-hexosaminidase improves diagnostic accuracy—from an area under the curve value of 0.72; with a sensitivity of 0.67; and specificity of 0.77 for only GCase activity, to an area under the curve value of 0.77; sensitivity, 0.71; and specificity, 0.85 for the combination in differentiating PD from healthy controls [61]. This improved diagnostic accuracy could be further increased by incorporating other markers that have similar behavior in differentiating PD from controls such as other lysosomal enzymes (acid sphingomyelinase, aSMase; acid-alpha galactosidase, GLA; acid alpha-glucosidase, GAA; galactocerebrosidase, GALC) and their relative activities, with and without association with each other [39].

Integrated biomarker set accuracy can also be further improved by incorporating markers from other generalist and specific pathways that represent the same trend of changes in CSF and/or serum as those seen in the PD brain such as 3-hydroxykynurenine [94,95], apolipoprotein D [96,97] and potentially neurofilament light chain [98]. With the same hypothesis, the diagnostic accuracy of multiple biochemical markers could be increased by combining them with imaging data. The data measured through different assays and research conditions could be combined by converting them to the percentage of changes (as seen in Table 1). Expressing metabolic changes as a percentage of changes would also allow different types of assays to be compared to each other. Finally, an iPD specific biomarker signature could be used for both differential diagnosis of iPD between its subtypes, and inform prognosis, considering the different weightings of each marker.

## 6. Conclusions

A definitive antemortem diagnosis of iPD remains an urgent and unaddressed need, especially during the prodromal stage. Several lines of clinical evidence on the diagnostic potential of GCase activity and its substrate accumulation in iPD are inconclusive; however, a growing interest in a comprehensive analysis of GCase activity in the context of the sphingolipid catabolism pathway is promising for non-invasive differentiation of iPD from healthy age-matched controls. Since PD is a multisystem disease with shared pathology with other atypical parkinsonism, future investigations with the integration of potential biomarkers from the sphingolipid pathway, imaging data and clinical symptoms could help improve iPD diagnostic accuracy in the clinic in early stages. Earlier diagnosis may also allow improved therapeutic interventions, where a wider therapeutic window for neuroprotection could be exploited.

## Figures and Tables

**Table 1:** Metabolic percentage of changes in sphingolipids pathway from postmortem PD brain are measured from CSF and/or blood compared to controls.

Pathway			Biomarker ID	Brain		CSF	Blood	References, Patient Population, Comments
				Other Parts	SN			
**Sphingolipid pathway**	**Sphingolipids**		Ceramide	−53% (ACC) (***), −26% (OC) (NS)		NS| +393% (***)	+5.48% (C16:0) (***), +3.34 (C18:0) (NS), +5.25% (C20:0) (**), 6.35% (C22:0) (**), +3.1% (C24:0) (NS), +4.2% (C26:0) (NS), +7.21% (C24:1) (**), +6.9% (C26:1) (*) [52] | −20.4% (C22:0) (***), −18.4% (C24:1) (***), −26.2% (C26:0) (***), −19.2% (C20:1) (**), −22.4 (C22:1) (***) [53]	**Brain:** Total ceramide in anterior cingulate cortex (ACC) and OC of iPD vs. HC [54] **CSF:** NS in PD-GBA vs. PD-GBA _wild-type_ [37,55] and PD-GBA _wild-type vs._ HC [55], increased in postmortem CSF of PD vs. HC [56] **Plasma:** PD-GBA _wild-type vs._ HC [52,53]
			Sphingomyelin			−0.4% (****)|+0.98% (*)|+200% (***)	+6.9% (C20:1) (**)	**CSF:** −0.4% in PD-GBA vs. HC [55], +0.98% in PD-GBA _wild-type_ vs. HC [37], +200% in in postmortem CSF of PD vs. HC [56] **Plasma:** PD-GBA _wild-type vs._ HC [53]
		**Glycosphingolipids**	Monosialoganglioside GM1a	−27% (**)	−22% in 70s (*), NS in 80s (1st cohort), −26% in 80s (*) (2nd cohort)| −73% (***)	−17% NS	−23% (**)| −32% (****), −57% (****), −37% (***)	**Brain (OC):** in iPD vs. HC [57] **SN:** in iPD vs. HC [40], −73% of GM1 positive dopaminergic neurons in iPD vs. HC [58] **CSF:** iPD vs. HC [40] **Serum:** −23% iPD vs. HC [40] **PBMC:** −32% in PD-GBA _wild-type_ vs. HC, −57% in PD-GBA vs. HC, −37% in PD-GBA vs. PD-GBA _wild-type_ [38]
			Ganglioside GD1a	−28% (**)	−39% in 70s (*), NS in 80s (1st cohort), NS in 80s (2nd cohort)	−38% (**)	−20% (**)	**Brain (OC):** in iPD vs. HC [57] **SN & CSF:** in iPD vs. HC [40] **Serum:** in iPD vs. HC [40]
			Ganglioside GD3			−33% (*)		**Brain (OC):** in iPD vs. HC [57] **SN & CSF:** in iPD vs. HC [40]
			Ganglioside GD1b	−12% (*)	−16% in 70s (*) −21% in 80s (*) (1st cohort), −31% in 80s (*) (2nd cohort)	−42% (***)		
			Ganglioside GT1b	NS	−23% NS in 70s −27% in 80s (*) (1st cohort), −34% in 80s (*) (2nd cohort)	−51% (***)		
			Ganglioside GM2			+23% (*)	−15% NS	**CSF & Serum:** in iPD vs. HC [40]
			Ganglioside GM3			+40% (*)	−8% NS|+14.5% (***)	**CSF:** in iPD vs. HC [40] **Serum:** NS in iPD vs. HC [40] **Plasma:** +14.5% in PD-GBA _wild-type vs._ HC [52]
			Gangliosides (sum of GM1a, GD1a, GD1b and GT1b)		−71% in 70s (**) NS in 80s (1st cohort), −67% in 80s (**) (2nd cohort)	−61% (**)		**SN & CSF:** in iPD vs. HC [40]
			Lactosylceramide (LacCer)		NS (1st cohort), NS (2nd cohort)	+22% (***)	NS| +2% (C16:0) (**), +4.8% (C18:0) (**), +4.5% (C22:0) (***), +4.4% (C24:0) (**), +4.7% (C24:1) (**)	**SN:** in NS in iPD vs. HC [40] **CSF:** in iPD vs. HC [40] **Serum:** NS in iPD vs. HC [40] **Plasma:** in PD-GBA _wild-type vs._ HC [52]
			Total glycosphingolipids (GlcCer, LacCer and gangliosides)		+31% NS in 70s +65% in 80s (***) (1st cohort), +39% in 80s (*) (2nd cohort)			**SN:** in iPD vs. HC [40]
			C18-Sphingosine		+86% in 70s (*), NS in 80s			
			Sphinganine		+87% in 70s (*), NS in 80s			
			Glucosylsphingosine	NS (Putamen)	+16% NS in 70s +116% (**) in 80s [40]| +77% in 60s (*), NS in 70s and 80s [59]			**Brain:** in PD-GBA _wild-type vs._ HC [59] **SN:** C18-glucosylsphingosine in iPD vs. HC [40], in PD-GBA _wild-type vs._ HC [59]
			Glucosylceramide		+37% NS in 70s +74% (***) in 80s, +45% in 80s (*) (2nd cohort)	NS, +18% (****)	NS [40]| +7.5% (***) (C16:0), +6% (*) (C18:0), +4.3 (*) (C20:0), +5.5 (***) (C22:0), +5.7% (**) (C24:0), NS (C26:0, C16:1, C22:1, C24:1)	**SN:** in iPD vs. HC [40] **CSF:** NS in PD-GBA _vs._ HC, +18% in PD-GBA vs. HC [55] **Serum:** NS in iPD vs. HC [40] **Plasma:** Monohexosylceramides in PD-GBA _wild-type vs._ HC [52]
	**Enzyme Activity**	GCase		−49% (*) in 60s, −44% (*) in 70s (Putamen) [59]| −20% (*) (Caudate) [35]	−34% in 70s (****), −26% in 80s (**) (1st cohort), −79% in 80s (*) (2nd cohort) [40]| −47% in 60e, NS in 70s and 80s [59]| −14% (*) [35]	NS| −28% (***)	−28% (*), −29% (*)	**Brain:** in PD vs. HC [35], in PD-GBA _wild-type vs._ HC [59]. **SN:** in PD-GBA and iPD [36], in iPD at their 60s not in 70s or 80s [59], in PD vs. HC [35] **CSF:** NS in PD vs. HC [37,60], −28% in PD vs. HC [61] **Blood (Monocytes):** in PD vs. HC (−28%), in PD-GBA _wild-type vs._ HC _wild-type_ (−29%). NS in lymphocytes [62]
		β-galactosidase		−0.3% NS	−71% in 70s (*), −77% in 80s (2nd cohort)| −11% NS	+37% (**)		**Brain (Caudate):** in PD vs. HC [35] **SN:** in iPD vs. HC [40], NS in PD vs. HC [35] **CSF:** in PD vs. HC [60]
		α-galactosidase			−59% in 70s (****), −65% in 80s (***) (1st cohort), −28% in 80s (*) (2nd cohort)		−7% (*), −9% (*)	**SN:** in iPD vs. HC [40] **Blood:** Dried blood spots from PD vs. HC [39]. −9% in iPD-GBA _wild-type_ over 40 age of onset vs. HC [39].
		β-hexosaminidase		−4.1% NS (Caudate)	−31% in 80s (**) (1st cohort), −23% (**) (2nd cohort)| +4.5% NS	−9% NS| −8% NS		**Brain tissue:** NS in PD vs. HC [35] **SN:** in iPD vs. HC [40], NS in PD vs. HC [35] **CSF:** −9% in PD vs. HC [61], −8% in PD vs. HC [60]
		Neuraminidase/Sialidase			−42% NS in 70s, −52% in 80s (*) (1st cohort), −54% in 80s (*) (2nd cohort)			**SN:** in iPD vs. HC [40]

Table legend: This table lists percentage changes of the sphingolipid pathway lipids and enzymes in the PD brain that are also measured in CSF and/or blood. The percentages are either reported by authors or calculated by considering the mean/median value of control data as the initial value. The percentage changes are increased (+) or decreased (−) metabolites levels in the PD cases compared to controls. The degree of significance is retrieved from sources. Abbreviations: HC: healthy control; PD: Parkinson’s Disease; PD-GBA: PD patients with GBA mutations; CSF: cerebrospinal fluid; SN: substantia nigra; OC: occipital cortex; GBA: Glucosylceramidase Beta1; |: separating data from different studies. (NS: non-significant; * *p* < 0.05; ** *p* < 0.01; *** *p* < 0.001; **** *p* < 0.0001).
